# Supplementary material for: Developing evidence for building sanitation justice: A multi methods approach to understanding public restroom quantity, quality, accessibility, and user experiences
Source: PLoS One. 2023 Jul 13;18(7):e0288525. doi: 10.1371/journal.pone.0288525 (PMC10343041; doi:10.1371/journal.pone.0288525)
Supplement: S1 Appendix — (DOCX) [file pone.0288525.s001.docx]

**Researcher Names (list at least 2 Research Assistants)**

**

**

**Enter date of assessment. mm/dd/yyyy**

**

**

**Enter time of assessment.**

**

**

**Restroom name/location/address: make sure to use the name reflected in the ESRI app**

**

**

**Unique Numeric ID: 4 digit ID reflected in the ESRI app**

**

**

**What facility type best describes this restroom?**

- **Public Building**
- **Outdoor Facility**
- **Government Facility**
- **Transportation**
- **Commercial**
- **Other**

**Select the public building sub category that best describes this restroom.**

- **Library**
- **Recreation Center**
- **Pools**
- **Senior Center**
- **Community Center**
- **Health Center**
- **Museum**

**Select the outdoor facility sub category that best describes this restroom.**

- **Park**
- **Beach**
- **Trail**
- **Campground**
- **Golf Course**

**Select the government facility sub category that best describes this restroom.**

- **Portland Loo**
- **City Building**
- **Police/Fire**
- **Stations Civic Center**

**Select the transportation sub category that best describes this restroom.**

- **Bus Stop**
- **Trolley Stop**
- **Gas Station**
- **Rest Stop**

**Select the commercial sub category that best describes this restroom.**

- **Shopping Center**
- **Restaurant**

**If “other” was selected, describe the facility type.**

**

**

**How many restroom buildings are at this site?**

- **1**
- **2**
- **3**
- **4**
- **>4**

**What are the hours of access?**

**

**

**Is this restroom open?**

- **Yes**
- **No**
- **Other**

**

**

**Is there a sign indicating where the nearest restroom is?**

- **Yes**
- **No**
- **Other

**

**How far is the nearest restroom?**

**

**

**Do you have any notes to add for this section?**

**

**

**Basic Features**

**Gender**

- **Female**
- **Male**
- **All gender**
- **Other**

**Are lights functioning?**

- **Yes**
- **No**
- **Other**

**Are sinks functioning?**

- **Yes**
- **No**
- **Other**

**Do the sinks have touch or touchless faucets?**

- **Touch faucets**
- **Touchless faucets**
- **Both**
- **Other**
- **None**

**Is there hot water?**

- **Yes**
- **No**
- **Other**

**How long for hot water?**

- **<10 seconds**
- **10-20 seconds**
- **21-30 seconds**
- **>30 seconds Other**

**Is there a hand dryer or paper towels?**

- **Hand dryer**
- **Paper towels**
- **Both**
- **Other**
- **None**

**Are there mirrors?**

- **Yes**
- **No**
- **Other**

**Is there a trashcan within the restroom?**

- **Yes**
- **No**
- **Other**

**How many toilets are there?**

- **1**
- **2**
- **3**
- **4**
- **5**
- **>5**

**How many urinals are there?**

- **1**
- **2**
- **3**
- **4**
- **5**
- **>5**
- **Nourinals**
- **Does not apply**

**Are any toilets broken/not functional?**

- **Yes**
- **No**
- **Other**

**How many toilets are broken?**

- **1**
- **2**
- **3**
- **4**
- **5**
- **>5**

**Please describe.**

**

**

**Are there doors?**

- **Yes**
- **No**
- **Other**

**Do the stall doors lock?**

- **Yes**
- **No**
- **Other**

**How is the bathroom being ventilated?**

- **Windows (high)**
- **Windows (barred)**
- **Fans**
- **Possible AC unit**
- **Open air ventilation**
- **No ventilation**
- **Other

**

**Are baby changing tables present?**

- **Yes**
- **No**
- **Other**

**Are the baby changing tables functioning?**

- **Yes**
- **No**
- **Other**

**Is there an ADA stall?**

- **Yes**
- **No**
- **Other**

**Do you have any notes to add for this section?**

**

**

**Health and Hygiene**

**Check off each hygiene product you see.**

- **Toilet paper**
- **Paper towel and/or hand dryers (duplicate) Soap**
- **Menstrual products**
- **Seat covers**
- **Did not assess**
- **Other**

**Any extra health or hygiene products available?**

- **Yes**
- **No**
- **Other**

**Please select the extra health or hygiene products available.**

- **Condoms**
- **Masks**
- **Hand sanitizer**
- **Other

**
- **No extra health or hygiene products**

**Is there a shower?**

- **Yes**
- **No**
- **Other**

**Select the type of shower.**

- **Indoor**
- **Outdoor**
- **Foot rinse station**
- **Other**

**Is there a safe needle deposit box?**

- **Yes**
- **No**
- **Other**

**Do you have any notes to add for this section?**

**

**

**External**

**Was this restroom easy to find/locate?**

**

**

**Is there a transit station nearby?**

- **Yes**
- **No**
- **Other**

**How close is the transit station?**

**

**

**Is there a bike rack nearby?**

- **Yes**
- **No**
- **Other**

**Is there a water fountain or bottle refill station nearby?**

- **Yes**
- **No**
- **Other**

**Are there security cameras? (if extra details, add to end of section notes)**

- **Yes**
- **No**

**What is the appearance around the restroom? (e.g. art, trees, restrooms by itself, trashcans, vending machines)**

**

**

**Were there people in/around the restroom when you were there? What were those people doing?**

**

**

**Are there any features that make the restroom look unsanitary? (e.g. wet floor, garbage can full, taped off toilet seat, yellow tape, scratched off mirror**

**

**

**Are there any barriers to access not noted? (e.g. police presence, ask for restroom to be unlocked)**

**

**

**Overall impressions/other notes**

**

**
