# Supplementary material for: Developing evidence for building sanitation justice: A multi methods approach to understanding public restroom quantity, quality, accessibility, and user experiences
Source: PLoS One. 2023 Jul 13;18(7):e0288525. doi: 10.1371/journal.pone.0288525 (PMC10343041; doi:10.1371/journal.pone.0288525)
Supplement: Attachment — Submitted filename: Response to Reviewers.docx [file pone.0288525.s002.docx]

Dear Editorial Team and Reviewers,

Thank you for the opportunity to revise and resubmit our manuscript [PONE-D-23–2687] entitled “Developing evidence for building sanitation justice: A multi methods approach to understanding public restroom quantity, quality, accessibility, and user experiences,” for publication in *PLOS ONE.* We thank the editorial team and the two reviewers for their helpful feedback, which we believe has strengthened the manuscript. We have provided responses to each of the comments below. Review comments are in bolded text and our responses are directly below.

**1. Please ensure that your manuscript meets PLOS ONE's style requirements, including those for file naming.**

We have double-checked the manuscript against the PLOS ONE style template and requirements and have made associated edits throughout.

**2. In the ethics statement in the Methods, you have specified that verbal consent was obtained. Please provide additional details regarding how this consent was documented and witnessed, and state whether this was approved by the IRB.**

We have expanded our description of the IRB approval for this study in Section 2.3 of the manuscript. We have provided an amended ethics statement in our cover letter and have also included it here: *This work was reviewed by the San Diego State University Institutional Review Board and determined to be a public health surveillance activity exempt from review. For the survey, the research team obtained comprehensive oral informed consent from each participant, and this was witnessed by two members of the research team. In order to preserve the anonymity of participants, we did not obtain written consent (signatures). Participants received a copy of the written consent document to keep, along with a comprehensive list of local resources (e.g., for housing, food, health care, and social services).*

**3. Thank you for stating the following financial disclosure: "The authors received support from the San Diego State University (SDSU) Emergency Funding for Student Assistance with Research, Scholarship, and Creative Activities (RSCA) Award (2021); the SDSU Summer Undergraduate Research Program (2021, 2022); SDSU’s Rapid Response Grants program; SDSU’s Seed Grant program; and SDSU’s School of Public Affairs. No grant numbers available. "**

**Please state what role the funders took in the study. If the funders had no role, please state: "The funders had no role in study design, data collection and analysis, decision to publish, or preparation of the manuscript." If this statement is not correct you must amend it as needed. Please include this amended Role of Funder statement in your cover letter; we will change the online submission form on your behalf.**

The funders had no role in study design, data collection and analysis, decision to publish, or preparation of the manuscript. We have provided an amended financial disclosure statement in the cover letter. The amended statement is also included here: *The authors received support from the San Diego State University (SDSU) Emergency Funding for Student Assistance with Research, Scholarship, and Creative Activities (RSCA) Award (2021); the SDSU Summer Undergraduate Research Program (2021, 2022); SDSU’s Rapid Response Grants program; SDSU’s Seed Grant program; and SDSU’s School of Public Affairs. No grant numbers available. The funders had no role in study design, data collection and analysis, decision to publish, or preparation of the manuscript. We have added this amended Role of Funder statement to the cover letter.*

**4. We note that you have indicated that data from this study are available upon request. PLOS only allows data to be available upon request if there are legal or ethical restrictions on sharing data publicly. For more information on unacceptable data access restrictions, please see** [**http://journals.plos.org/plosone/s/data-availability#loc-unacceptable-data-access-restrictions**](http://journals.plos.org/plosone/s/data-availability#loc-unacceptable-data-access-restrictions)**.**

We have provided an amended data availability statement in the cover letter. The amended statement is also provided here: *Public restroom location data and short-form assessment data are freely available at bathrooms.sdsu.edu and are provided in the manuscript. Due to ethical, privacy, and safety concerns for the respondents of our social survey, we do not share a de-identified dataset of social survey responses. People experiencing homelessness are an extremely vulnerable population. Many unhoused people experience daily the threat of criminalization simply by existing in public space. Developing trust and protecting privacy are two critical elements of conducting research with and on behalf of unhoused people and the organizations who serve them. We asked sensitive questions regarding where people go to use the restroom, related health concerns and priorities, etc. While unlikely, these data could feasibly be used to identify an individual respondent. Please contact the SDSU Institutional Review Board with any concerns: irb@sdsu.edu*

**5. Please include your full ethics statement in the ‘Methods’ section of your manuscript file. In your statement, please include the full name of the IRB or ethics committee who approved or waived your study, as well as whether or not you obtained informed written or verbal consent. If consent was waived for your study, please include this information in your statement as well.**

We have expanded our description of the IRB approval for this study in Methods Section 2.3 of the manuscript and provided all details related to our ethics approval.

**6. We note that Figures 3 and 4 in your submission contain [map/satellite] images which may be copyrighted. All PLOS content is published under the Creative Commons Attribution License (CC BY 4.0), which means that the manuscript, images, and Supporting Information files will be freely available online, and any third party is permitted to access, download, copy, distribute, and use these materials in any way, even commercially, with proper attribution. For these reasons, we cannot publish previously copyrighted maps or satellite images created using proprietary data, such as Google software (Google Maps, Street View, and Earth). For more information, see our copyright guidelines:** [**http://journals.plos.org/plosone/s/licenses-and-copyright**](http://journals.plos.org/plosone/s/licenses-and-copyright)**.**

Figures 3 and 4 have been edited to use the OpenStreetMap basemap which is licensed under a Creative Commons License. Additional details on OpenStreetMap CC BY 4.0 licensing is available at this link: <https://www.esri.com/arcgis-blog/products/arcgis-online/mapping/whats-new-with-openstreetmap-basemap-march-2019/>

We have included the reviewer comments in bolded text below with our response immediately following.

**Reviewer #1: The manuscript has focused on a serious issue on Public Health and Hygiene, at San Diego, US. Authors have described that they have developed three tools, "public restroom spatial database, field assessment tool, and survey, and document process to collect these public restroom data in a consistent, public health and equity-driven framework".**

**Although the strategy of the tool development has been explained in the manuscript, it has not explained the languages/codes used for tool and database development.**

We have added additional information about the softwares used for tool and database development. These additions were made in Sections 2.1 and 2.3. These changes include a description of the use of Microsoft Excel and Qualtrics. We have also clarified the process through which restroom locations are documented and mapped using latitude and longitude.

**In methods section, Tool 1, page 179, there is a mention of 669 datapoints (public restrooms). This data is converted into a map and made public on ArcGIS online. The ArcGIS online URL is missing. Not sure whether the 669 datapoints (raw data) has been shown in the above-mentioned website.**

The restroom location data are available on ArcGIS Online. We have clarified the data availability and appropriate citation. The URL for our online map has beed added to the text. This URL is <https://bathrooms.sdsu.edu/>. We have also improved the image resolution in Figure 3, showing a static image of our ArcGIS Online web map.

**The main source of the data repository (**[**https://bathrooms.sdsu.edu/**](https://bathrooms.sdsu.edu/)**) is not mentioned explicitly in the manuscript text or abstract. Only mentioned in the last but second figure, (that is also with poor resolution). Without highlighting this data source, explicity, it is diffcult to understand the consequences of the results.**

The data repository URL is now included in Section 2.1 of the manuscript, above Figure 3. The resolution of Figure 3 has also been improved.

**There are four figures mentione in the text. However, those are very difficult to trace, because those four figures attached at the end of the mansucript do not contain any labels (although Figure legends are mentioned within the text). Moreover, the qualities (resolutions) of the figures are very poor. The texts embedded in the figures, specifically, the last two are almost illegible.**

Figure titles are included in the uploaded file names in accordance with PLOS ONE style requirements. We have improved the image resolution for all figures in the manuscript.

**Overall the work addressed a serious public issue worth to be considered and put forward for action, it requires minor changes before it can be considered for acceptance.**

Reviewer #1, we thank you very much for your review of the manuscript.

**Reviewer #2: This article aims to address the very critical issues related to sanitation security among PEH group in San Diego. The methods undertaken and analysis part is also appropriate. But, the word limit is seems to be more and presentation style requires more concise, clear meaning to the reader and requires major revision. There are typo and punctuation error and formatting correction is required ( for example Sentence 71 is not completed; Sentence 81, more explanation the meaning of e.g. Flush-Toilet Finder (22) and Toilet Finder (23) is required etc).**

We have done a complete read through of the manuscript to ensure concise, clear language throughout. The sentence in line 71 has been checked for completeness and the sentence in line 81 has been clarified with additional text: *While there remains no strategic policy approach to public toilet provision (21), some crowd-sourced, online databases of public restrooms have been developed to try to fill this void (e.g. Flush-Toilet Finder (22) and Toilet Finder (23)). Both Flush-Toilet Finder and Toilet Finder are web-based mobile phone applications with information about public restroom locations.*

**The limitations of the study should be clearly highlighted.**

We have expanded our discussion of study limitations in the conclusion (pp. 29-30). We note that the topical contributions of this work are limited to people experiencing homelessness and staying in the Pacific Beach neighborhood in San Diego. Findings from this population may not be generalizable to other marginalized populations or other geographic contexts.

**The IRB approval for the study must be taken.**

Additional information about the IRB review and approval of this work has been added to page 12, Section 2.3 of the manuscript. The San Diego State University Institutional Review Board reviewed all research activities reported in the manuscript and deemed them exempt from on-going review. For the survey, the research team obtained comprehensive oral informed consent from each participant, and this was witnessed by two members of the research team. In order to preserve the anonymity of participants, we did not obtain written consent (signatures). Participants received a copy of the written consent document to keep, along with a comprehensive list of local resources (e.g., for housing, food, health care, and social services).

**Since there are three methodological tool has been developed for sanitation justice, all technical concepts related to methodological development must be addressed.**

We have added additional information about the softwares used for tool and database development. These additions were made in Sections 2.1 and 2.3. We have also further detailed our restroom inclusion criteria on page 8-9.

**Also detailed description of data triangulation for case study approach needs to be highlighted.**

Additional information on methods triangulation has been added to Section 3.4 on p. 23 as well as in the Discussion on p. 29 – we added additional examples of the unique and specific knowledge gained from using these three tools together. We have included the following text to the Discussion section:

*Lastly, methods triangulation allowed us to corroborate findings, identify tensions and inconsistencies, and to increase the validity of our findings. In one critical example of this – hours of operation – our social survey respondents confirmed what we suspected but were unable to verify during neither our spatial database development nor our field assessment. More than half of survey respondents reported experiencing restricted access to their main restroom due to limited hours of operation and/or unsanitary conditions, with two-thirds of respondents reporting a public restroom being too far away or no public restrooms nearby where they stay (67%, n=30). Further, while our academic research team’s field assessment data suggested that PB restrooms were well-stocked, social survey respondents reporting on their lifetime history with public restrooms clarify that the provision of key resources like soap and toilet paper are in fact highly variable. Also, hot water emerged as a critical and valued resource for the unhoused population that was often absent at public restrooms.*

**The key findings in the conclusion section need to be tightly built up.**

The Discussion section (pp. 26-31) has been edited to highlight key methodological and topical takeaways from this work. We include the following:

*In this paper, we document both the methodological contributions of this work and highlight topical findings from a case study in San Diego, California. Together, these contributions provide new methods to investigate and understand public restroom availability and quality in a study region and individuals’ experiences with restroom (in)access.*

*By using our tools in concert with one another, we are able to holistically document and assess access to and conditions of public restrooms in a given region, as well as the lived experiences and health needs related to restroom access of a population highly reliant on public restrooms (unhoused peoplePEH). Together, the three tools extend prior research to offer layered insight into the true conditions of the sanitation landscape. The spatial database development provides a macro-level baseline understanding of the number and distribution of restrooms in the built environment. The tool offers a systematic approach to documenting public restroom availability where previous efforts have relied on incomplete administrative and crowd-sourced datasets. Restroom spatial database development is one approach to understanding gaps in public restroom availability and can serve to broaden public information on availability facilities. In addition, as demonstrated in the case study, the spatial database can be linked to other existing data to understand issues of sanitation inequity and the relationship between the availability of sanitation resources and health-related needs on a broad scale.*

**Hope the reviewer comments would be helpful to strengthen the manuscript.**

Reviewer #2, we thank you very much for your review of the manuscript.

Thank you all again for your feedback and we look forward to hearing from you.
